# First Detection and Genomic Characterization of Feline Orthopneumovirus From Domestic Cats in South Korea

**DOI:** 10.1155/tbed/7077703

**Published:** 2025-09-18

**Authors:** Jonghyun Park, Seung-Chun Park, Choi-Kyu Park, Hye-Ryung Kim

**Affiliations:** ^1^College of Veterinary Medicine and Institute for Veterinary Biomedical Science, Kyungpook National University, Daegu, Republic of Korea; ^2^Laboratory of Veterinary Pharmacokinetics and Pharmacodynamics, College of Veterinary Medicine and Cardiovascular Research Institute, Kyungpook National University, Daegu, Republic of Korea; ^3^DIVA Bio Incorporation, Iksan 54531, Republic of Korea

**Keywords:** feline, molecular analysis, *Orthopneumovirus*, phylogenetic analysis, South Korea

## Abstract

Orthopneumoviruses have been found in humans and various animal hosts, including mice, cattle, dogs, and swine, in several countries, but have rarely been found in cats, with the only report being from the United States in 2010. This study is the first to detect feline orthopneumovirus (FPnV) in domestic cats in South Korea and the first to characterize the complete genomic sequence of the virus worldwide. FPnV was detected in 7 of 318 feline respiratory clinical samples, resulting in a detection rate of 2.2%. A complete genome sequence and a G gene sequence were successfully obtained from two FPnV-positive samples. Sequence analysis of these Korean FPnV strains (KFPnV-2201 and KFPnV-2202) showed the highest homology with the Korean swine orthopneumovirus (SOV) strain, KSOV-2201, which was recently identified in domestic pigs in South Korea. Surprisingly, KFPnVs showed relatively low homology with the FPnVs previously reported in the United States. As a result of phylogenetic analysis, FPnV strains previously reported in the United States were classified as genogroup 1, while two FPnV strains in Korea were classified as genogroup 2, along with four strains from the United States and Korea, and two canine orthopneumovirus (CPnV) strains in China. These results suggest that genetically diverse FPnV strains may be widely distributed globally, highlighting the need for continuous surveillance of the virus. Additionally, the high genetic homology among the viruses derived from different hosts, including cats, dogs, and pigs, suggests the possibility of cross-species transmission. These findings provide evidence that genetically diverse orthopneumoviruses are circulating in various animal hosts and that these viruses may be evolving through cross-species transmission. Therefore, further extensive studies are needed to understand the epidemiology, pathogenesis, and genetic evolution of FPnV.

## 1. Introduction

The family *Pneumoviridae* comprises large, enveloped, negative-sense RNA viruses and is classified into two genera: *Orthopneumovirus* and *Metapneumovirus*. Currently, three viral species have been classified within the genus *Orthopneumovirus*: *Orthopneumovirus hominis* (formerly known as human respiratory syncytial virus, HRSV); *Orthopneumovirus bovis* (formerly known as bovine respiratory syncytial virus, BRSV); and *Orthopneumovirus muris* (formerly known as murine pneumonia virus, MPV) [1]. The genomic organization of orthopneumoviruses is different from that of metapneumoviruses and is arranged as follows: nonstructural protein 1 (NS1)-NS2-nucleocapsid protein (N)-phosphoprotein (P)-matrix protein (M)-small hydrophobic protein (SH)-attachment glycoprotein (G)-fusion protein (F)-M2-large polymerase (L) [1, 2]. The G protein gene plays a critical role in viral infection and pathogenesis and is commonly used for the genetic characterization and phylogenetic analysis of orthopneumovirus [3, 4].

Recently, viruses genetically and phylogenetically related to MPV have been identified in various animal hosts, including dogs, cats, and pigs. Canine orthopneumovirus (CPnV) was initially identified in the United States from canine respiratory samples collected between 2008 and 2011 [4–6]. Subsequently, it has been identified in Europe [2, 7], Asia [8], and Oceania [9]. Swine orthopneumovirus (SOV) was first identified in the United States [10] and later in France [11], South Korea [3], and Germany [12]. Unlike CPnV and SOV, feline orthopneumovirus (FPnV) was identified in the respiratory tracts of cats in the United States in 2010 [6] but has not been reported in any other countries. These three orthopneumoviruses remain unclassified within the genus due to limited genetic and virological information. Despite the difference in animal hosts, these viruses exhibit over 90% homology in their complete genome sequences and share greater similarity with MPV than with HRSV and BRSV [3]. Therefore, for convenience, these viruses are collectively referred to as MPV-like orthopneumoviruses (MLOVs), consistent with a previous study [3]. Previous studies have demonstrated that MLOVs form a distinct phylogenetic clade separate from MPVs [6, 8]. A recent comprehensive analysis of all available MLOV strains classified these viruses into two genogroups, G1 and G2 [3]. The two FPnV strains previously reported in the United States both belonged to genogroup G1, showing close genetic relationships with CPnV strains.

However, unlike CPnV or SOV, FPnV has not been identified in countries outside the United States to date. This may be due to FPnV not being considered a major feline respiratory pathogen and consequently being excluded from routine diagnostic panels for feline respiratory pathogens. The present study aimed to conduct molecular surveillance to determine whether FPnV has been introduced into the Korean cat population. Here, we report the first detection and characterization of FPnV in domestic cats in South Korea. Sequence analysis was performed to characterize the Korean FPnV strain and trace its potential origin.

## 2. Materials and Methods

### 2.1. Sample Collection and RNA Extraction

A total of 318 nasopharyngeal samples were collected from domestic cats with respiratory symptoms in South Korea. These samples were voluntarily submitted to the College of Veterinary Medicine and the Institute for Veterinary Biomedical Science at Kyungpook National University from animal hospitals in South Korea as part of the feline respiratory disease surveillance project. Viral RNA was extracted from 200 μL of each sample using a TANBead nucleic acid extraction kit with a fully automated magnetic bead platform (Taiwan Advanced Nanotech Inc., Taoyuan, Taiwan). The extracted RNA was eluted with 100 μL of the elution buffer following the manufacturer's instructions. All nucleic acid samples were stored at −80°C until analysis.

### 2.2. Molecular Detection of FPnV and Other Feline Pathogens

To detect FPnV RNA, a real-time reverse transcription-polymerase chain reaction (qRT-PCR) assay was conducted using previously described primers (forward: 5′-AAGATAAATTCTTCTATGAAAACAGAATGA-3′; reverse: 5′-CCATCATAAGTGAGATTTCTAT-3′) and a probe (5′-6-carboxyfluorescein-CTGCCTAAGTACTATCCAGCCATACTGC-Black Hole Quencher 1–3′) targeting the conserved M2-1 and M2-2 genes of MPVs and MLOVs [2, 3]. The qRT-PCR was performed with 0.4 µM of each primer and 0.2 µM of the probe using a commercial qRT-PCR kit (THUNDERBIRD Probe One-step qRT-PCR kit, TOYOBO, Osaka, Japan) with the CFX96 Touch Real-Time PCR Detection System (Bio-Rad, Hercules, CA, USA). To confirm the coinfection status of FPnV-positive samples, five feline respiratory pathogens, including feline calicivirus (FCV), feline herpesvirus 1 (FHV-1), *Bordetella bronchiseptica*, *Mycoplasma felis*, and *Chlamydia felis*, were tested using previously described qPCR and qRT-PCR assays [13, 14]. Nuclease-free water was included as a negative control from the nucleic acid extraction step, and negative controls showed no amplification in all qRT-PCR reactions.

### 2.3. Genome Sequencing of FPnV

To genetically characterize the Korean FPnV strains, complete genomes and G gene sequences were analyzed using FPnV-positive samples obtained in this study. The primer sets used for sequencing were previously reported primer sets designed based on the complete genome sequences of MPV, SOV, and CPnV [3]. Briefly, cDNA fragments were synthesized using the PrimeScript 1st Strand cDNA Synthesis Kit (Takara Korea Biomedical Inc., Seoul, Republic of Korea). PCR was performed with the synthesized cDNA and the specified primer sets using the PrimeSTAR GXL DNA Polymerase Kit (Takara Korea Biomedical Inc.) according to the manufacturer's instructions. The amplified PCR products were purified using the GeneAll Expin Combo GP 200 Miniprep Kit (GeneAll, Seoul, Republic of Korea). The sequences of each product were analyzed in duplicate using BIONICS (Daejeon, Republic of Korea) based on Sanger's method. The complete genome sequence (KFPnV-2201) and G gene sequence (KFPnV-2202) were successfully obtained from two FPnV-positive samples with low Ct values (≤27) and deposited in the GenBank database under the accession numbers PQ381743 and PQ381744, respectively.

### 2.4. Multiple Alignment and Phylogenetic Analysis

For comparative analysis, 12 complete genome sequences (2 MPVs, 7 CPnVs, and 3 SOVs) and 17 G gene sequences (14 CPnVs, 2 FPnVs, and 1 SOV) were obtained from GenBank (accessed on March 30, 2024). The reference sequences for BRSV (GenBank Accession: NC_038272), HRSV type A (GenBank Accession: NC_038235), and HRSV type B (GenBank Accession: MW582529) were used as outgroups for the complete genome analysis. The list of sequences used in the analysis is provided in Table S1. Multiple sequence alignments were generated using MAFFT [15], available in Geneious Prime (https://www.geneious.com accessed on March 30, 2024). For the phylogenetic analysis, the IQ-TREE 2 software package (http://www.iqtree.org accessed on April 15, 2024) was used [16]. The best-fit substitution model (TIM2+F+I+R2) was selected using ModelFinder (https://www.mathworks.com/help/simulink/slref/modelfinder.html; accessed on April 15, 2024) [17], and a maximum likelihood phylogenetic tree was constructed through ultrafast bootstrap analysis with 1000 replicates [18]. The phylogenetic tree was visualized using the iTOL phylogenetic tree viewer [19].

### 2.5. Virus Isolation Attempts

The isolation attempt of FPnV was performed according to previous studies [4]. Briefly, A72 cells were cultured in Dulbecco's Modified Eagle Medium (D-MEM; Gibco Co., New York, NY, USA) supplemented with 10% heat-inactivated fetal bovine serum (FBS; Gibco Co., New York, NY, USA) and 1% antibiotic–antimycotic (Anti–Anti; Gibco Co., New York, NY, USA). FPnV-positive samples were then inoculated onto monolayer A72 cells (CRL-1542, American Type Culture Collection, Manassas, VA, USA) cultures. The cell cultures were maintained through subculturing every 5 days, with this process repeated for a total of three passages. During each passage, qRT-PCR was performed, and in the third passage, immunofluorescence staining was performed using a monoclonal antibody specific to HRSV (Invitrogen, RSV3216).

## 3. Results

### 3.1. Detection of FPnV and Other Feline Respiratory Pathogens

During 2022, a total of 318 samples were collected from cats exhibiting respiratory symptoms. These included 124 samples (39.0%) from cats aged 1–12 months, 29 samples (9.1%) from cats aged 12–24 months, 16 samples (5.0%) from cats aged 24–36 months, 13 samples (4.1%) from cats aged 36–48 months, 20 samples (6.3%) from cats aged 48–60 months, and 114 samples (35.8%) from cats over 60 months, while the age of 2 samples was unknown. Analysis of 318 feline samples using the qRT-PCR assay identified 7 samples positive for FPnV, resulting in a detection rate of 2.2%. The FPnV-positive cats represented six distinct breeds, with ages ranging from 2 to 71 months. Notably, most (5/7) of FPnV-positive cats were under 6 months of age. Details of FPnV-positive samples are presented in Table 1. To assess coinfection status, the FPnV-positive samples underwent additional molecular screening for other common feline respiratory pathogens, including FCV, FHV-1, Bb, Mf, and Cf. Among the seven FPnV-positive samples, four exhibited coinfection with one or more of these pathogens, while three samples were positive for FPnV alone (Table 1).

### 3.2. Genome Sequencing of Korean FPnVs

In this study, we successfully obtained the complete genome sequence of strain KFPnV-2201 (GenBank Accession Number PQ381743) and the G gene sequence of strain KFPnV-2202 (GenBank Accession Number PQ381744) from samples F262 and F67, respectively (Table 1). The complete genome sequence of the KFPnV-2201 strain was 14,879 bp in length and consisted of 11 open reading frames encoding NS1–NS2–N–P–M–SH–G–F–M2-1–M2-2–L, consistent with previously reported orthopneumoviruses [2, 4].

**Figure 1:**
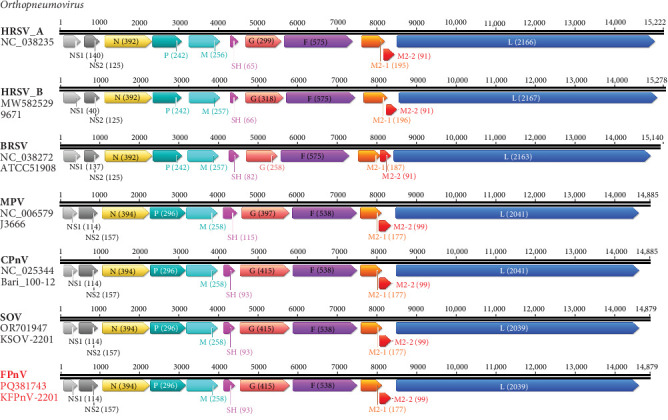
Genome organization of representative members of the genus *Orthopneumovirus*. The complete genome sequences of each virus are expressed in the sense (coding) direction (3′–5′). The black lines represent the nucleotide length of each genome, with scale bars indicating nucleotide positions. Each box, drawn to scale, represents an mRNA gene corresponding to a separate coding region and contains the abbreviation of the corresponding gene, with the amino acid sequence length indicated in parentheses. The boxes corresponding to each gene are represented in different colors: nonstructural protein 1 (NS1), light gray; NS2, dark gray; nucleocapsid protein (N), light yellow; phosphoprotein (P), teal; matrix protein (M), sky blue; small hydrophobic protein (SH), pink; attachment glycoprotein (G), light red; fusion protein (F), purple; M2-1, orange; M2-2, dark red; large polymerase (L), blue. On the left side of each gene organization diagram, information about the virus is indicated in the order of the virus name, GenBank number, and strain name. The strain name of KFPnV-2201, identified in this study, is represented in red.

As shown in Figure 1, the genomic length of the KFPnV-2201 strain was similar to that of MPV and MLOV strains, including CPnV and SOV, differing by only six nucleotides (nts). However, it was substantially shorter than the reference HRSV type A, HRSV type B, and BRSV strains, with a difference of 343, 399, and 261 nts, respectively. These length variations were attributed to cumulative differences in individual gene lengths. For the SH proteins, HRSV type A, HRSV type B, BRSV, and MPV have lengths of 65, 66, 82, and 115 aa, respectively, while MLOVs have a length of 93 aa. The G proteins, known to be involved in viral cell attachment, have lengths of 299, 318, 258, and 397 aa in HRSV type A, HRSV type B, BRSV, and MPV, respectively, while MLOVs have a longer length of 415 aa. The L proteins in MLOVs, which function as RNA-dependent RNA polymerases (RdRPs), were similar to MPV with a length of 2041 amino acids, but shorter than those of HRSV type A, HRSV type B, or BRSV. The results showed that the KFPnV-2201 strain was highly related to the MPV and MLOV strains derived from murine, canine, and swine origins but distantly related to other orthopneumovirus strains derived from human and bovine sources.

### 3.3. Phylogenetic Analyses of Korean FPnVs

**Figure 2:**
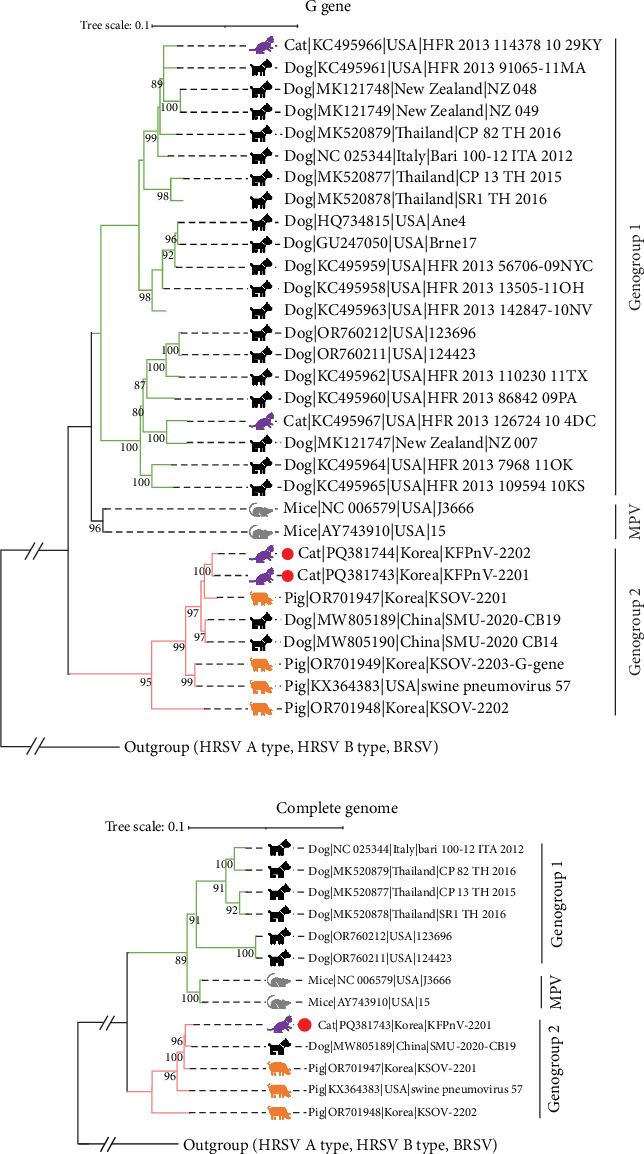
Phylogenetic trees based on G gene (A) and complete genome (B) sequences of Korean feline orthopneumovirus. The phylogenetic tree was constructed using 34 G genes and 16 complete genome sequences, including the two KFPnVs identified in this study (red circles). BRSV (GenBank Accession: NC_038272), HRSV type A (GenBank Accession: NC_038235), and HRSV type B (GenBank Accession: MW582529) sequences were used as outgroup sequences for each phylogenetic tree. The positions of murine pneumoviruses (MPVs) and genotype 1 and genotype 2 of MPV-like orthopneumoviruses (MLOVs) were located on the right side of each phylogenetic tree. Bootstrap values greater than 90 are indicated with Arabic numerals in the corresponding branches. Scale bars indicate nucleotide substitutions per site.

Phylogenetic analyses were conducted using 16 complete genome sequences and 34 G gene sequences of orthopneumoviruses, including the KFPnV-2201 and KFPnV-2202 strains identified in this study (Figure 2). The phylogenetic tree based on G gene sequences revealed that MPV and MLOV strains, excluding the three outgroup strains (HRSV A type, HRSV B type, and BRSV), clustered into two distinct genogroups: genogroup 1 (G1) and genogroup 2 (G2). KFPnV-2201 and KFPnV-2202 clustered within G2 MLOV, showing close genetic relationships to SOV strains from South Korea (KSOV-2201, KSOV-2202, and KSOV-2203) and the United States (SOV 57), as well as to the Chinese CPnV strains (SMU-2020-CB19 and SMU-2020-CB14) (Figure 2A). The phylogenetic analysis based on the complete genome sequences corroborated the G gene-based analysis, confirming that KFPnV-2201 belongs to G2, clustering with SOV and CPnV strains from South Korea, the United States, and China (Figure 2B).

### 3.4. Genetic Characterization of Korean FPnVs

To assess the genetic homology of orthopneumoviruses, the complete genome sequence of the KFPnV-2201 strain was compared with those of 10 MLOV strains, including seven CPnV and three SOV, and five non-MLOV strains, including two MPV, one HRSV type A, one HRSV type B, and one BRSV (Table 2). The complete genome sequence of KFPnV-2201 exhibited high homology with MPVs and MLOVs, with nt sequence identities of 92.2%–92.3% and 90.0%–99.1%, respectively. In contrast, it showed low homology with HRSV A, HRSV B, and BRSV, with nt sequence identities of 47.1%, 47.5%, and 47.2%, respectively. The KFPnV-2201 genome showed higher homology to MPV (92.2%–92.3%) than to G1 MLOVs (90.0%–90.6%) and displayed the highest homology with G2 MLOVs (97.3%–99.1%). Among G2 MLOVs, KFPnV-2201 shared the highest nt sequence identity (99.1%) with the swine-origin KSOV-2201 strain. It also showed high homology with the U.S. SOV strain SOV 57 (98.0%) and the Chinese CPnV strain SMU-2020-CB19 (97.3%). It also showed relatively high homology with the SOV strain SOV 57, previously identified in the United States, and the CPnV strain SMU-2020-CB19, previously identified in China, with nt sequence identities of 98.0% and 97.3%, respectively. When the individual gene sequences of KFPnV-2201 and four G2 MLOV strains were compared, the NS1 and M genes of KFPnV-2201 exhibited the highest homology with their counterparts in KSOV-2201. The NS2, N, P, G, and L genes demonstrated the highest similarity to the corresponding genes of the SMU-2020-CB19 strain. Meanwhile, the SH and M2-1 genes showed the greatest homology with their counterparts in SOV 57.

Additionally, a G gene sequence of KFPnV-2202 was successfully obtained from the F67 sample. The G gene sequences of KFPnV-2201 and KFPnV-2202 were compared with those of 32 orthopneumovirus strains currently available (Table 3). The G genes of the two Korean FPnVs showed the highest homology with each other, with 99.5% nt sequence identity. They also exhibited relatively high homology with 27 MLOVs and two MPVs with 85.9%–98.8% and 89.0%–89.3% nt sequence identities, respectively. In contrast, low homology was observed with HRSV A type, HRSV B type, and BRSV with 25.7%–25.9%, 28.7%–28.9%, and 26.8%–27.0% identities, respectively (Table 3). The two Korean FPnV strains are closely related to G2 MLOVs, including three SOV strains (SOV 57 from the United States; KSOV-2201, KSOV-2202, and KSOV-2203 from South Korea) and two CPnVs (SMU-2020-CB19 and SMU-2020-CB-14 strains from China), with 97.3–98.8 nt sequence identities. However, they are distantly related to the previously reported FPnV strains (114378_10_29KY and 91065-11MA strains from the United States), with 86.9%–87.5% nt sequence identities.

## 4. Discussion

Since its first report in the United States in 2010 [6], FPnV had not been identified in any other countries until this study. This study identified FPnV for the second time globally and the first time in South Korean cats, with a prevalence of 2.2%. The ages of the FPnV-positive cats varied from 2 to 71 months, with most (5 out of 7) being kittens under 6 months old. Importantly, the kittens' samples had lower Ct values than those from older FPnV-positive cats aged 14 and 71 months. These findings suggest that kittens may be more susceptible to FPnV infection or may be in environments where they are more likely to be exposed to the virus. Kittens are often raised in groups at pet shops or breeding facilities before being newly adopted into household environments. In such cases, the higher positivity rate and relatively lower Ct values observed in kittens may reflect transmission within their original group setting rather than intrinsic age-related susceptibility. A limitation of this study was the inability to analyze environmental and epidemiological factors, such as adoption history or housing conditions. To further elucidate the viral characteristics and epidemiology of FPnV, investigations targeting facilities that supply kittens are warranted.

Out of seven FPnV-positive cats, four were coinfected with other feline respiratory pathogens, while the other three were infected solely with FPnV. These results suggest that FPnV may cause respiratory symptoms in cats, particularly in young kittens, either through a single infection of the virus or coinfection with other respiratory pathogens. In this regard, it is noteworthy that CPnV is considered an emerging respiratory pathogen associated with the canine respiratory disease complex (CRDC) and is currently included in the list of etiological diagnoses of CRDC [2, 20, 21]. Therefore, further studies are needed to determine the prevalence of FPnV in cats with respiratory symptoms and to elucidate its potential role in the pathogenesis of the feline respiratory disease complex (FRDC) through virus isolation.

Despite the presence of sufficient viral nucleic acid in clinical samples to obtain complete genome sequences, attempts to isolate Korean FPnV in vitro were unsuccessful. No results for virus isolation were obtained from qRT-PCR analysis and immunofluorescence analysis. The primary reason for this failure is likely the use of previously collected samples rather than fresh, active specimens. Additionally, previous research has shown that even within the same cell line, different clones may express varying levels of cellular receptors [2]. The host cell receptor for FPnV has not yet been identified. Further research on the FPnV receptor is necessary not only for establishing cell lines suitable for virus isolation but also for predicting host range and elucidating the virus's infection mechanism.

The genus *Orthopneumovirus*, within the family *Pneumoviridae*, comprises only three classified virus species [1]. Among these, *Orthopneumovirus muris* (MPV) stands apart from the closely related *Orthopneumovirus hominis* (HRSV) and *Orthopneumovirus Bovis* (BRSV). Previous reports and our study demonstrate significant genetic divergence between MPV and the RSV species [2, 5]. While the complete genome sequences of HRSV and BRSV share approximately 94% identity, they exhibit only about 60% similarity with that of MPV [5]. Recently, viruses similar to MPV have been detected in pigs, dogs, and cats [2–10, 12, 21]. Complete genome sequences of these viruses, elucidated through this study and previous research [2, 5, 10], show more than 90% homology. However, these viruses have not yet been officially classified and are referred to by different names depending on the host. In this study, we used the designation “MLOV”, consistent with previous research findings, but formal classification is needed in the future.

To genetically characterize KFPnV-2201 and KFPnV-2202, the viral sequences of FPnV-positive samples were analyzed, and a complete genomic sequence of KFPnV-2201 and a G gene sequence of KFPnV-2202 were successfully obtained. To the best of our knowledge, this is the first report of a complete genome sequence of FPnV, as only two G gene sequences of FPnV strains from the United States have been reported to date. The complete genome sequence length and composition of coding proteins in FPnV were similar to other MLOVs and MPV. However, differences in amino acid lengths of proteins, including G and L, compared to BRSV or HRSV suggest these viruses may possess distinct characteristics. Complete genome sequence analysis revealed that the genome homology of each protein-coding region in FPnV was less than 60% compared to HRSV or BRSV, with the G protein showing less than 30% homology. This suggests that while individual proteins of MLOVs may exhibit general characteristics of pneumoviruses, their mechanisms of action on the host could differ from those of HRSV and BRSV. Further research is needed to elucidate these differences.

Phylogenetic analysis and genomic characterization based on the G gene and complete genome sequences have provided valuable insights into the classification and evolutionary dynamics of MLOV strains. Korean FPnV strains belong to G2 and demonstrate the highest homology with four SOV strains (KSOV-2201, KSOV-2202, and KSOV-2203 from South Korea; SOV 57 from the United States) and two CPnVs (SMU-2020-CB19 and SMU-2020-CB-14 from China). Notably, Korean FPnVs showed the highest homology (99.1%) with the KSOV-2201 strain identified from a Korean pig farm [3]. These findings suggest that Korean FPnVs may be undergoing evolutionary adaptation through cross-species transmission while circulating between pigs and cats in South Korea. Unexpectedly, KFPnV-2201 and KFPnV-2202 showed relatively low homology with FPnVs previously reported in the United States (114378_10_29KY and 91065-11MA) [4]. The two FPnV strains previously reported in the United States were classified under G1, suggesting a different evolutionary relationship between Korean FPnV strains classified as G2. The differentiation of FPnV strains from the two countries into distinct genotypes suggests that geographical factors may have a greater impact on the genetic diversity of FPnV than host factors. This observation underscores the genetic diversity of FPnVs and highlights the need for investigations into FPnVs across multiple countries to confirm potential transboundary transmission of MLOVs.

The G2 MLOVs derived from three hosts in three countries highlight the necessity of monitoring these viruses across various host species. Previous studies have indicated the potential risk of cross-species transmission of orthopneumoviruses among different host species [2, 3, 10]. Negative-strand RNA viruses require transcription of their genomic RNA into positive-sense strands prior to replication, exhibiting heightened reliance on host cellular machinery due to compact genome size. Their error-prone replication, driven by rapid replication cycles and absent polymerase proofreading, enables mutation rates up to 10^5^-fold higher than DNA viruses, serving as critical host-adaptation drivers for zoonotic spillover [22]. Current evidence suggests that MLOV exhibits broad host adaptability and zoonotic potential. However, no studies have systematically characterized virological determinants such as receptor-binding affinity, host-cell entry mechanisms, or immune evasion strategies. Future studies focused on MLOV could provide critical data for both veterinary virology and public health.

## 5. Conclusion

This study reports the first identification of FPnV in domestic cats with respiratory symptoms in South Korea. Genetic and phylogenetic analyses revealed that these Korean FPnV strains were genetically distinct from previously reported FPnVs in the United States. Notably, they showed the closest relatedness to SOV and CPnV recently identified in Korean pigs and Chinese dogs, respectively. This genetic similarity suggests the potential for cross-species transmission of these viruses. These findings provide valuable insights into the genetic diversity and evolution of FPnVs, underscoring the importance of continuous surveillance of MLOVs across various animal hosts. Further research is warranted to elucidate the epidemiology, pathogenesis, and genetic evolution of FPnVs.

## Figures and Tables

**Table 1:** Characteristics of the FPnV-positive samples and detection of FPnV and other feline respiratory pathogens.

Sample number	Breeds	Age (months)	FPnV detection (Ct value)	Strain sequenced	Coinfection with other respiratory pathogens
F67	Selkirk rex	2	26.65	KFPnV-2202	Bb, Mf
F107	Munchkin	2	28.51	—	FCV, Bb
F186	Korean shorthair	71	36.70	—	ND
F197	Norwegian forest	3	27.93	—	ND
F251	Korean shorthair	14	31.20	—	FCV, FHV-1, Cf
F262	Ragdoll	6	24.53	KFPnV-2201	ND
F279	British shorthair	3	28.97	—	FCV

Abbreviations: Bb, *Bordetella bronchiseptica*; Cf, *Chlamydia felis*; FCV, feline calicivirus; FHV-1, feline herpesvirus-1; FPnV, feline orthopneumovirus; Mf, *Mycoplasma felis*; ND, not detected.

**Table 2:** Comparison of the complete genome sequences between KFPnV-2201 and other orthopneumovirus strains.

Host	Virus/ genotype	Strain	Nucleotide identity (%) to KFPnV-2201											
			Complete	NS1	NS2	N	P	M	SH	G	F	M2-1	M2-2	L
Canine	MLOV/G1	Bari/100-12	90.6	89.2	85.4	92.9	92.2	93.3	91.8	87.4	92.3	93.0	92.9	92.4
Canine	MLOV/G1	CP_13_TH	90.6	89.2	86.8	92.6	92.0	93.2	92.8	86.8	92.2	93.0	93.3	92.3
Canine	MLOV/G1	CP_82_TH	90.2	88.0	85.4	92.4	91.4	92.5	91.8	87.2	91.5	93.0	92.9	92.2
Canine	MLOV/G1	SR1_TH	90.4	88.3	86.2	92.6	92.1	93.2	92.8	86.4	92.1	92.8	93.3	92.2
Canine	MLOV/G1	123696	90.0	90.9	86.4	92.0	90.8	93.0	91.0	86.2	91.2	92.7	94.3	91.9
Canine	MLOV/G1	124423	90.0	91.2	86.4	92.0	90.8	93.0	91.0	86.2	91.2	92.7	94.3	91.9
Canine	MLOV/G2	SMU-2020-CB19	97.3	97.7	98.7	99.4	99.2	99.6	97.8	98.8	99.6	98.7	99.3	99.5
Swine	MLOV/G2	KSOV-2201	99.1	98.8	98.1	99.2	98.6	99.7	98.2	98.6	99.6	99.1	99.3	99.4
Swine	MLOV/G2	KSOV-2202	95.4	96.8	93.8	95.6	95.2	97.2	94.6	93.5	96.8	97.0	96.0	96.3
Swine	MLOV/G2	57	98.0	93.3	97.7	98.7	98.4	99.2	98.6	97.5	98.8	99.2	99.0	99.1
Murine	MPV	J3666	92.3	93.9	88.5	94.2	93.5	95.0	92.8	89.3	93.7	93.8	94.3	93.6
Murine	MPV	mice_15	92.2	93.9	88.5	94.2	93.1	94.6	90.0	89.2	93.6	93.8	94.3	93.6
Human	HRSV/A	M74568	47.1	29.2	29.4	60.2	41.6	54.1	36.2	25.7	48.4	54.0	32.8	54.6
Human	HRSV/B	9671	47.5	29.7	29.4	60.3	41.4	52.6	33.3	26.6	49.4	52.7	31.0	55.2
Bovine	BRSV	ATCC51908	47.2	31.4	30.8	59.8	40.8	53.6	30.7	23.1	49.8	52.1	30.1	54.8

*Note*: F, fusion protein; G, attachment glycoprotein; L, large polymerase; M, matrix protein; N, nucleocapsid protein; NS, nonstructural protein; P, phosphoprotein; SH, small hydrophobic protein.

Abbreviations: BRSV, bovine respiratory syncytial virus; HRSV, human respiratory syncytial virus; MLOV, MPV-like orthopneumovirus; MPV, murine pneumonia virus.

**Table 3:** Nucleotide sequence homology of the G genes of KFPnV-2201 and KFPnV-2202, with other orthopneumovirus strains.

Number	Host	Virus/ genotype	Strain	GenBank number	Percentage of identity to KFPnV-2201 (%)	Percentage of identity to KFPnV-2202 (%)	Number	Host	Virus (genotype)	Strain	GenBank number	Percentage of identity to KFPnV-2201 (%)	Percentage of identity to KFPnV-2202 (%)
1	Feline	MLOV/G2	KFPnV-2201	PQ381743	—	99.5	18	Canine	MLOV/G1	110230-11TX	KC495962	86.3	86.0
2	Feline	MLOV/G2	KFPnV-2202	PQ381744	99.5	—	19	Canine	MLOV/G1	142847-10NV	KC495963	87.8	87.5
3	Swine	MLOV/G2	KSOV-2201	OR701947	98.6	98.2	20	Canine	MLOV/G1	7968-11OK	KC495964	87.2	86.7
4	Swine	MLOV/G2	KSOV-2202	OR701948	93.5	93.2	21	Canine	MLOV/G1	109594-10KS	KC495965	87.6	87.3
5	Swine	MLOV/G2	KSOV-2203	OR701949	97.5	97.3	22	Canine	MLOV/G1	124423	OR760211	86.2	85.9
6	Swine	MLOV/G2	57	KX364383	97.5	97.3	23	Canine	MLOV/G1	123696	OR760212	86.2	85.9
7	Canine	MLOV/G2	SMU-2020-CB-14	MW805190	98.8	98.5	24	Canine	MLOV/G1	CP13-TH-2015	MK520877	86.8	86.5
8	Canine	MLOV/G2	SMU-2020-CB19	MW805189	98.8	98.5	25	Canine	MLOV/G1	SR1-TH-2016	MK520878	86.4	86.1
9	Feline	MLOV/G1	114378_10_29KY	KC495966	87.5	87.1	26	Canine	MLOV/G1	CP82-TH-2016	MK520879	87.2	86.9
10	Feline	MLOV/G1	91065-11MA	KC495967	87.2	86.9	27	Canine	MLOV/G1	NZ_007	MK121747	86.2	85.9
11	Canine	MLOV/G1	Bari_100_12	NC_025344	87.4	87.1	28	Canine	MLOV/G1	NZ_048	MK121748	86.9	86.7
12	Canine	MLOV/G1	Ane4	HQ734815	87.6	87.3	29	Canine	MLOV/G1	NZ_049	MK121749	86.9	86.7
13	Canine	MLOV/G1	Bme17	GU247050	87.5	87.1	30	Murine	MPV	J3666	NC_006579	89.3	89.0
14	Canine	MLOV/G1	13505-11OH	KC495958	87.1	86.8	31	Murine	MPV	15	AY743910	89.2	89.2
15	Canine	MLOV/G1	56706-09NYC	KC495959	87.6	87.2	32	Human	HRSV/A	HRSV_A_type	NC_038235	25.9	25.7
16	Canine	MLOV/G1	86842-09PA	KC495960	87.5	87.3	33	Human	HRSV/B	9671	MW582529	28.9	28.7
17	Canine	MLOV/G1	91065-11MA	KC495961	87.6	87.3	34	Bovine	BRSV	ATCC51908	NC_038272	27.0	26.8

Abbreviations: BRSV, bovine respiratory syncytial virus; HRSV, human respiratory syncytial virus; MLOV, MPV-like orthopneumovirus; MPV, murine pneumonia virus.

## Data Availability

The datasets supporting the conclusions of this article are included within the article and its additional files. The complete genome sequence and G gene sequence of Korean FPnVs obtained in this study were submitted to the GenBank database (Accession Numbers: PQ381743 for KFPnV-2201 and PQ381744 for KFPnV-2202).

## Supporting Information

Supporting Information Table S1. Information on sequences of orthopneumoviruses used in this study.
