# Supplementary material for: First Detection and Genomic Characterization of Feline Orthopneumovirus From Domestic Cats in South Korea
Source: Transbound Emerg Dis. 2025 Sep 18;2025:7077703. doi: 10.1155/tbed/7077703 (PMC12463527; doi:10.1155/tbed/7077703)
Supplement: Supporting Information — Table S1. Information on sequences of orthopneumoviruses used in this study. [file 7077703.f1.docx]

**Supplementary data**

**Table S1. Information on Sequences of Orthopneumoviruses used in this study**

| No. | Host | Strain | Country | Collection  year | GenBank accession number | Sequence^a^  (base-pairs) |
| --- | --- | --- | --- | --- | --- | --- |
| 1 | Feline | KFPnV-2201^b^ | South Korea | 2022 | PQ381743 | Complete (14887) |
| 2 | Feline | KFPnV-2202^b^ | South Korea | 2022 | PQ381744 | G (1245) |
| 3 | Feline | 114378-10-29KY | United States | 2013 | KC495966 | G (1245) |
| 4 | Feline | 126724-10-4DC | United States | 2013 | KC495967 | G (1260) |
| 5 | Murine | 15 | United States | 1939 | AY743910 | Complete (14887) |
| 6 | Murine | J3666*^c^* | United States | - | NC_006579 | Complete (14885) |
| 7 | Swine | 57 | United States | 2012 | KX364383 | Complete (14885) |
| 8 | Swine | KSOV-2201 | South Korea | 2022 | OR701947 | Complete (14879) |
| 9 | Swine | KSOV-2202 | South Korea | 2022 | OR701948 | Complete (14885) |
| 10 | Swine | KSOV-2203 | South Korea | 2022 | OR701949 | G (1245) |
| 11 | Canine | Ane4 | United States | 2008 | HQ734815 | Partial (8598) |
| 12 | Canine | Bme17 | United States | 2008 | GU247050 | Partial (10963) |
| 13 | Canine | 56706-09NYC | United States | 2009 | KC495959 | G (1245) |
| 14 | Canine | 86842-09PA | United States | 2009 | KC495960 | G (1245) |
| 15 | Canine | 142847-10NV | United States | 2010 | KC495963 | G (1245) |
| 16 | Canine | 109594-10KS | United States | 2010 | KC495965 | G (1245) |
| 17 | Canine | 13505-11OH | United States | 2011 | KC495958 | G (1245) |
| 18 | Canine | 91065-11MA | United States | 2011 | KC495961 | G (1245) |
| 19 | Canine | 110230-11TX | United States | 2011 | KC495962 | G (1245) |
| 20 | Canine | 7968-11OK | United States | 2011 | KC495964 | G (1245) |
| 21 | Canine | dog/Bari/100-12/ITA/2012 | Italy | 2012 | NC_025344 | Complete (14885) |
| 22 | Canine | NZ_007 | New Zealand | 2014 | MK121747 | G (1266) |
| 23 | Canine | NZ_048 | New Zealand | 2015 | MK121748 | G (1245) |
| 24 | Canine | NZ_049 | New Zealand | 2015 | MK121749 | G (1245) |
| 25 | Canine | CP13-TH-2015 | Thailand | 2015 | MK520877 | Complete (14793) |
| 26 | Canine | SR1-TH-2016 | Thailand | 2016 | MK520878 | Complete (14789) |
| 27 | Canine | CP82-TH-2016 | Thailand | 2016 | MK520879 | Complete (14790) |
| 28 | Canine | SMU-2020-CB19 | China | 2020 | MW805189 | Complete (14786) |
| 29 | Canine | SMU-2020-CB14 | China | 2020 | MW805190 | Partial (8266) |
| 30 | Canine | USA/LA/2022/124423 | United States | 2022 | OR760211 | Complete (14832) |
| 31 | Canine | USA/LA/2022/123696 | United States | 2022 | OR760212 | Complete (14786) |
| 32 | Human | HRSV_type_A | United States | 1987 | NC_038235 | Complete (15222) |
| 33 | Human | HRSV type B_9671 | Germany | 2015 | MW582529 | Complete (15278) |
| 34 | Bovine | BRSV_ ATCC51908 | United States | 2000 | NC_038272 | Complete (15140) |

^a^ Complete, complete genome sequence; G, G gene sequence; Partial, partial gene sequence including G gene sequence.

^b^ Feline orthopneumovirus strains sequenced in this study.

^c^ Strains 15 and J3666 were both isolated at the Rockefeller Institute, New York, and are believed to be descended from the same original virus isolate.
